# Effect of triple inhaled therapy on MACE and cardiovascular events in COPD: a systematic review and meta-analysis

**DOI:** 10.3389/fcvm.2025.1680080

**Published:** 2025-12-04

**Authors:** A. Calderón-Montero, J. de Miguel Díez, V. Barrios, C. Calderón-Ferrer, M. Joshi

**Affiliations:** 1 Cerro del Aire Primary Care Health Center, SERMAS, Madrid, Majadahonda, Spain; 2 Pneumology Department, Gregorio Marañon Hospital, Madrid, Spain; 3 Cardiology Department, Ramón y Cajal Hospital, Madrid, Spain; 4 Degree in Chemistry Complutense University, Madrid, Spain; 5 Independent Chartered Statistician, Birmingham, United Kingdom

**Keywords:** MACE, cardiovascular events, triple inhaled therapy, COPD, meta-analysis

## Abstract

**Background:**

Although some meta-analyses show that triple inhaled therapy (TT) reduces all-cause mortality compared with dual inhaled therapy (DT), the effect on cardiovascular events is not yet well defined. We estimated the effect of TT compared with DT (LAMA/LABA or LABA/ICS) on MACE and cardiovascular outcomes in an evidence synthesis.

**Methods:**

Following prospective registration (https://osf.io/gtfvm), a comprehensive search strategy of PubMed, Scopus, and Embase was performed until 15 January 2025. All randomized clinical trials (RCTs) evaluating TT vs. DT and reporting MACE and cardiovascular outcomes were included. We assessed risk of bias and conducted a random-effects meta-analysis estimating summary relative risk (RR) with 95% confidence intervals, evaluating heterogeneity using *I*^2^. A network meta-analysis (NMA) was undertaken.

**Results:**

From 781 citations, five RCTs were selected (7,855 patients receiving TT, 7,003 LABA/ICS, 5,059 LAMA/LABA). The risk of bias was moderate in three and low in two RCTs. TT reduced MACE by a non-significant 11% vs. LAMA/LABA (0.89; 0.70–1.12, four RCTs, *I*^2^ = 0%) and increased by a non-significant 26% vs. LABA/ICS (1.26; 0.97–1.64, four RCTs, *I*^2^ = 0%). TT reduced cardiovascular mortality (CVD) by 50% (0.50; 0.31–0.80, three RCTs, *I*^2^ = 0%) and increased non-fatal stroke by 92% (1.92; 1.09–3.39, two RCTs, *I*^2^ = 0%) compared with LAMA/LABA. TT shows a favorable trend in myocardial ischemia outcomes. For CVD, NMA showed that TT ranked first and LAMA/LABA last in effectiveness.

**Conclusions:**

In exacerbating patients with moderate to very severe COPD, TT significantly reduces CVD compared with LAMA/LABA dual therapy, without a significant reduction in MACE.

**Systematic Review Registration:**

https://doi.org/10.17605/OSF.IO/GTFVM.

## Introduction

Recently, the Global Initiative for Chronic Obstructive Lung Disease (GOLD) 2025 guidelines have published their latest revision (1), which highlights the recommendation of triple inhaled therapy (TT) as the only inhaled pharmacological treatment indicated to reduce all-cause mortality. In addition, it highlights the importance of considering cardiovascular comorbidity in the diagnosis and management of chronic obstructive pulmonary disease (COPD), establishes the long-acting muscarinic antagonist/long-acting β2-agonist (LAMA/LABA) combination as the basis for the treatment of COPD, especially in non-exacerbators, and relegates the LABA/ICS (long-acting β2-agonist/inhaled glucocorticoids) combination to specific situations.

Cardiovascular disease is highly prevalent in COPD (2–4). The most recent clinical trials of TT show that cardiovascular events are the leading cause of mortality and morbidity in COPD, overcoming respiratory causes (5–8). However, the evidence on the effect of TT on cardiovascular events has not been specifically studied. In one study, TT suggests a reduction in cardiovascular mortality (CVD) (9), while other studies reported that the effect is neutral (10) or has not been adequately assessed (5, 6, 11). A specific clinical trial is currently under development to evaluate the effect of TT on cardiopulmonary complications with results to be published in 2028 (12).

In an extensive literature review, we did not find any meta-analyses specifically designed to study the effect of TT compared with dual inhaled therapy (DT) (LAMA/LABA or LABA/ICS) on the incidence of cardiovascular events in COPD. To clarify this aspect, we developed the present evidence synthesis for estimating the effect of TT compared with DT subtypes on cardiovascular events by evaluating the incidence of MACE and its components and other specific cardiovascular variables.

## Material and methods

### Prospective registration and design

This systematic review was conducted following prospective registration (Center for Open Science, on 16 April 2023; https://doi.org/10.17605/OSF.IO/GTFVM). The review followed the recommended methodology for collating and synthesizing all eligible randomized clinical trials (RCTs), and the manuscript was written using the recommended methodology for reporting systematic reviews and meta-analyses (13, 14).

### Literature search and selection

A comprehensive literature search strategy was employed covering databases PubMed, Scopus, and Embase, without language or time restrictions, until 10 January 2025. The final search combined the keywords and word variations of the following terms: “chronic obstructive pulmonary disease,” “cardiovascular mortality,” “myocardial infarction,” “stroke,” “cardiovascular events,” “triple inhaled therapy,” “dual inhaled therapy,” and “randomized clinical trials.” The complete search strategy is detailed in Supplementary Appendix S1. Mendeley software was used to manage retrieved citations.

To be considered eligible, studies should include the following criteria: randomized double-blind clinical trial assessing the effect of TT compared with any DT (LABA/LAMA or LABA/ICS), reporting MACE and its components and other cardiovascular outcomes, such as heart failure (HF) and myocardial ischemia, in patients with moderate to very severe COPD. Inhaled therapy (triple and dual) had to be administered in a single device and with a duration of 12 months as the minimum clinically relevant time to analyze fatal cardiovascular events (15). Table 1 lists each trial's inclusion criteria and whether a history of prior exacerbations was required.

**Table 1:** Summary of included studies in the meta-analysis, inclusion criteria, and sociodemographic and clinical characteristics of the population.

	KRONOS (11)	TRIBUTE (6)	TRILOGY (5)	IMPACT (7, 10)	ETHOS (8, 9)
Author	Ichinoise M	Papi A	Singh D	Lipson DA Day NC^a^	Rabe KF Martinez FJ^a^
Year	2019	2018	2016	2020	2021
Triple therapy (µg)	Budesonide/glycopyrrolate/formoterol, 320/18/9.6	Beclomethasone/glycopirronium/formoterol, 87/5/9	Beclomethasone/glycopirronium/formoterol, 100/6/12.5	Fluticasone/umeclidinium/vilanterol, 100/62.5/25	Budesonide/glycopyrrolate/formoterol, 320/18/9.6
Dual therapy (µg)	Formoterol/glycopyrrolate, 9.6/18 Budesonide/formoterol, 320/9.6	Indacaterol/glycopirronium, 85/43	Beclomethasone/formoterol, 100/6	Umeclidinium/vilanterol, 62.5/25 Fluticasone/vilanterol, 100/25	Glycopyrrolate/formoterol, 18/9.6 Budesonide/formoterol, 320/9.6
Key inclusion criteria	Eligible patients had a clinical history of COPD, a smoking history of ≥10 pack-years, COPD Assessment Test score ≥10, and a FEV1 ≥25% and <80%. There was no requirement for a history of COPD exacerbations in the year prior to study entry	Elegible patients had symptomatic COPD, severe or very severe airflow limitation, at least one moderate or severe exacerbation in the previous year, and were receiving inhaled maintenance medication	Patients with COPD and FEV1 lower than 50%, one or more moderate to severe COPD exacerbations in the previous 12 months, COPD Assessment Test total score ≥10, and a Baseline Dyspnea Index focal score of 10 or less	Eligible patients were 40 years or older, had to have either an FEV1 < 50% and a history of at least one moderate or severe exacerbation in the previous year, or an FEV1 of 50 to 80% and at least two moderate exacerbations or one severe exacerbation in the previous year	Eligible patients were 40–80 years old with symptomatic COPD, a FEV1 25%–65% of the predicted normal value, and a history of ≥1 moderate or severe COPD exacerbations in the previous year (if FEV1 was <50%) or ≥2 moderate or ≥1 severe COPD exacerbations (if FEV1 was ≥50%)

	KRONOS (11)	TRIBUTE (6)	TRILOGY (5)	IMPACT (7, 10)	ETHOS (8, 9)
Subjects (*N*)					
TT	116	764	687	4,151	2,137
LAMA/LABA	111	768		2,070	2,121
LABA/ICS	58		680	4,134	2,120
Smoker (%)	40/41/19	46/43	47/47	31/33/37	40/39/30
Follow-up^b^	52	52	52	52	52
Age (mean)	69.4/69.0/69.7	64.5/64.5	63.3/63.8	65.3/65.2/65.3	64.6/64.8/64.6
Male (%)	93/91/97	72/72	74/77	67/66/66	59/59/60
Prior severe exacerbations (%)	26/18/17	NA	NA	75/75/74	21/20/21
HTA (%)	NA	57/60	59/56	51/51/53	59/59/59
DM (%)	NA	13/14	13/13	15/15/16	19/16/19
Prior coronary disease (%)	NA	18/20	25/25	26/26/26	14/13/15
Prior heart failure	NA	10/10	11/13	5/6/5	NA

FEV1, post-bronchodilator forced expiratory volume in 1 s; NA, data not reported; HTA, hypertension; DM, diabetes mellitus.

When data is separated by “/,” the results are shown in the following order: triple therapy/comparator. When there are two comparators, the data correspond to the following order: triple therapy/LAMA/LABA combination/LABA/ICS combination.

a First author on substudies of mortality and cardiovascular complications.

b Folow-up in weeks.

**Figure 1:**
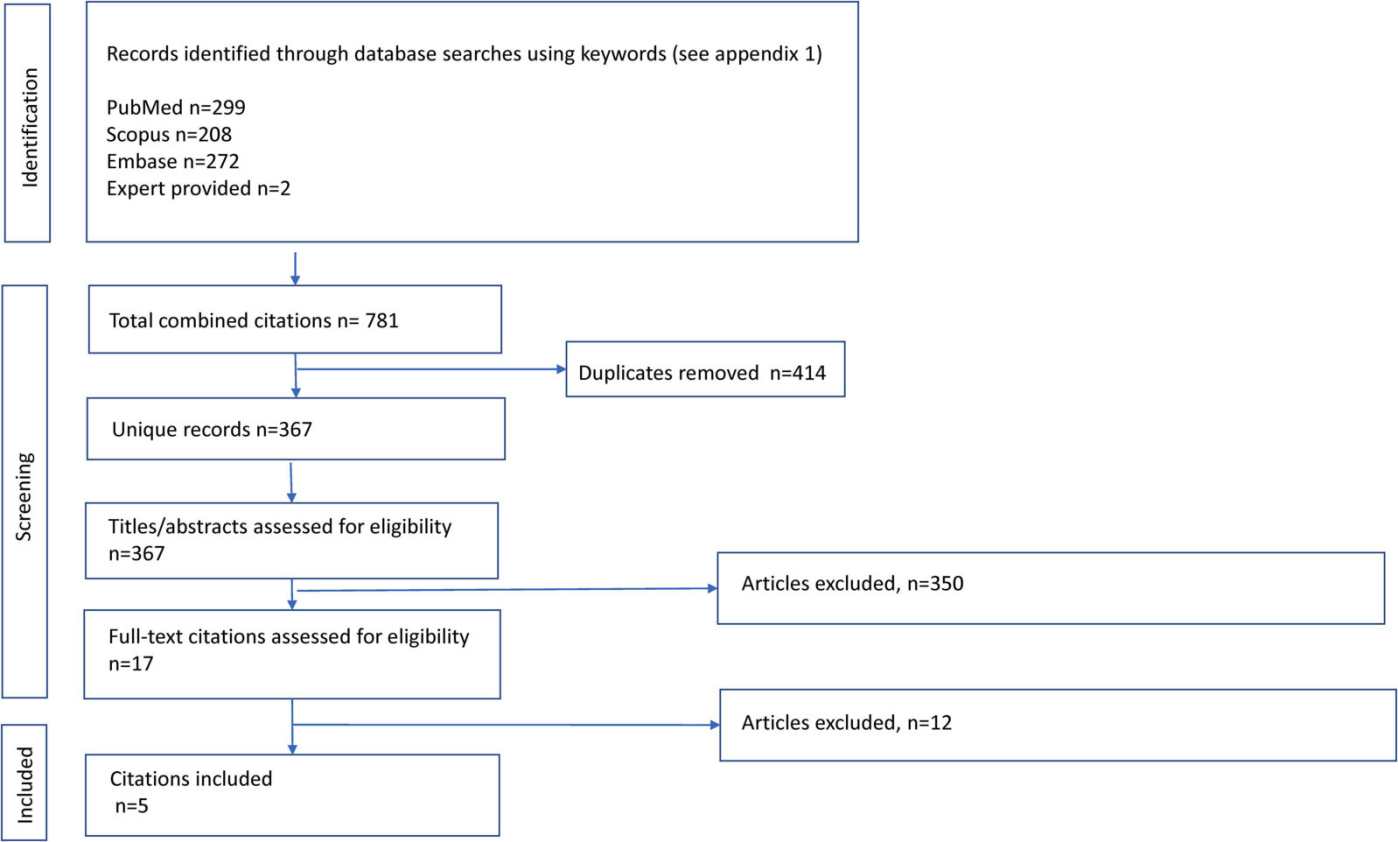
Flow diagram of study selection for the systematic review of inhaled therapy in chronic obstructive pulmonary disease.

Studies were selected through a multistep approach, including deletion of exact and inexact duplicates, reading of titles and abstracts, and assessment of full-texts performed independently by two investigators (Figure 1). To ensure interobserver variability, a pilot study was performed beforehand using 50 studies, which showed a 95% level of agreement. Initially, all the studies that met the inclusion criteria were selected, followed by the detection of duplicate studies and those that did not meet the eligibility criteria based on their titles and abstracts. Subsequently, with the remaining studies, the full manuscripts were analyzed in depth to discard those that did not strictly meet the selection criteria (Supplementary Appendix S2). Discrepancies between investigators were resolved by consensus by revising the original or by arbitration by a third reviewer.

### Data extraction and study quality assessment

Data were extracted independently by two investigators from the original publications. Data collected for the analysis included cardiovascular outcomes, such as major adverse cardiovascular events (MACE), cardiovascular death, non-fatal myocardial infarction, non-fatal stroke, myocardial ischemia, cardiac adverse events (CAE), cardiovascular adverse events of special interest (CVAESI), all HF, and non-fatal HF, which were considered the most relevant variables. In general, we chose on-treatment data rather than off-treatment data to specifically analyze the efficacy of treatments. Criteria for the selection and definition of cardiovascular outcomes are detailed in Supplementary Appendix S3. Included RCTs were critically appraised by two investigators using the risk-of-bias tool for randomized trials (RoB 2) (16). Data for the studies included in the meta-analysis were extracted both from the original publication (5–8, 11) and from the cardiovascular substudies (9, 10). In the case of the ETHOS trial (8), only the data for the budesonide 320 µg dose were used for analysis as this is the dose available in clinical practice (17).

### Data synthesis

The primary objective was to analyze the effect of TT compared with DT on MACE and its components as the primary endpoint. Simultaneously, CAE, myocardial ischemia, CVAESI, and HF were analyzed as secondary relevant endpoints. Analyses of all variables were performed for both DT (LAMA/LABA and LABA/ICS). Additionally, a comparison between LABA/ICS and LAMA/LABA was performed secondarily to assess the impact of inhaled corticosteroids on cardiovascular events as detailed in Supplementary Appendix S4.

Data on primary and secondary endpoints that are extracted separately from each included study on patients with COPD were used to estimate relative risks along with 95% confidence intervals (CI). The incidence of cardiovascular events was calculated from the original data and expressed per 1,000 patients/year. The analyses comparing TT and the two DT for MACE and cardiovascular events were conducted using a pairwise meta-analysis using a random-effects model. Our primary estimand was on-treatment exposure, although we prespecified complementary treatment policy/ITT analyses as sensitivity analyses. Heterogeneity among studies was assessed using the *Q* test and *I*-squared (*I*^2^) statistic. We assumed that *I*^2^ > 50% indicated substantial heterogeneity and *I*^2^ > 75% indicated considerable heterogeneity. We performed a subgroup analysis based on the different treatment types, LAMA/LABA and LABA/ICS, to identify potential sources of heterogeneity and analyze potential differences in the estimates according to subgroups. We planned to use funnel plots to detect potential reporting biases and evaluated the ranking of effectiveness using network meta-analysis (NMA) for MACE and its components, combining indirect comparisons where available with direct comparisons. Network meta-analyses were carried out using a common-effects model (Mantel–Haenszel method) to hierarchically rank the therapies using the netrank *P*-score (18). In addition, as part of the sensitivity analysis, a Bayesian NMA was carried out to evaluate the ranking using the surface under the cumulative ranking curve (SUCRA) statistic (19). All statistical analyses were conducted using R software.

## Results

### Characteristics of the selected studies

Of the total of 781 citations, 5 met the selection criteria for analysis (Figure 1). Briefly, the patients included (7,855 patients receiving TT, 7,003 LABA/ICS, and 5,059 LAMA/LABA) were adults older than 40 years, predominantly male, and smokers or ex-smokers with moderate to very severe COPD severity and 52 weeks of exposure to inhaled therapy. The characteristics of the studies included in the meta-analysis and of the patients are described in detail in Table 1.

#### Risk of bias

**Figure 2:**
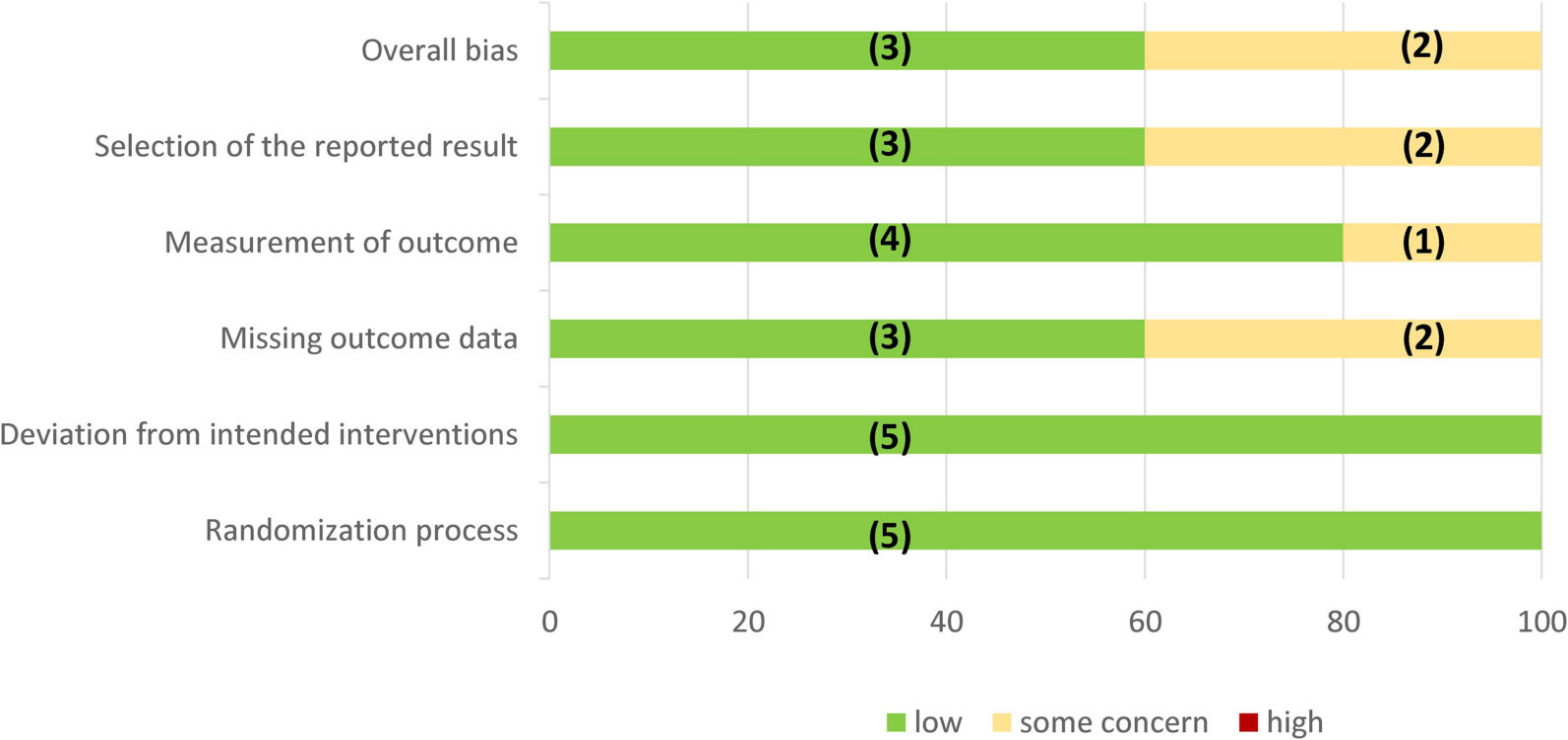
Assessment of RoB 2 bias risk by domain. In parentheses is the absolute number of studies.

Regarding the main outcome, all studies provided data on MACE, cardiovascular mortality, and non-fatal myocardial infarction, while in two studies (40%), no data on non-fatal stroke could be obtained (Figure 2). With respect to secondary outcomes, three out of five (60%) of the studies provided data on CAE, CVAESI, and HF. Of the five included studies, two (40%) showed a low risk of bias, while three were considered to be at moderate risk of bias (Supplementary Appendix S5).

### Outcome assessment

The main results of the meta-analysis are summarized in Table 2. TT was compared with the two dual therapies (LAMA/LABA and LABA/ICS) using a paired analysis of on-treatment patients. To provide a comprehensive overview of the effect of TT compared with both DT, Table 2 presents the relative risk, absolute and relative incidence of events, absolute risk, number of patients who must be treated for 1 year, and level of evidence using the GRADE methodology.

**Table 2:** Summary of findings of the main results obtained in the meta-analysis (on-treatment analysis).

Comparator	Outcome	Events/*N*		Relative risk	Rate^a^		ARR	NNT	GRADE
		TT	Control	TT vs. control	TT	Control			
LAMA/LABA	MACE	156/7,191	133/5,096	−11% (0.89, 0.70–1.12)	21.7	26.1	4.4	227	Low–moderate
	Cardiovascular death	32/6,427	46/4,328	−50% (0.50,0.31–0.80)	5.93	11.63	5.7	176	High–moderate
	Non-fatal MI	31/7,052	37/4,958	−39% (0.61, 0.33–1.10)	4.4	7.46	3.06	327	Moderate
	Non-fatal stroke	50/6,288	37/4,190	+92% (1.92, 1.09–3.39,	7.9	3.8	−4.1	NA	Low
LABA/ICS	MACE	127/7,114	136/7,084	+26% (1.26, 0.97–1.64)	21.7	14.1	−7.6	NA	Low–moderate
	Cardiovascular death	32/6,427	38/6,404	−16% (0.84, 0.52–1.34)	5.93	6.64	0.71	1,408	Moderate
	Non-fatal MI	31/7,114	29/7,084	−13% (0.87, 0.35–2.18)	4.36	4.10	−0.26	NA	Low
	Non-fatal stroke	50/6,288	27/6,265	+84% (1.84, 1.16–2.94)	7.9	4.3	−3.6	NA	Low

a Per 1,000 patients/year.

Pairwise meta-analyses used random-effects. NNT was calculated only when ARR > 0 (benefit).

MI, myocardial infarction; NNT, number of patients to be treated; NA, not applicable.

Below, we present detailed data for MACE and the main cardiovascular events analyzed.

#### MACE and its components

**Figure 3:**
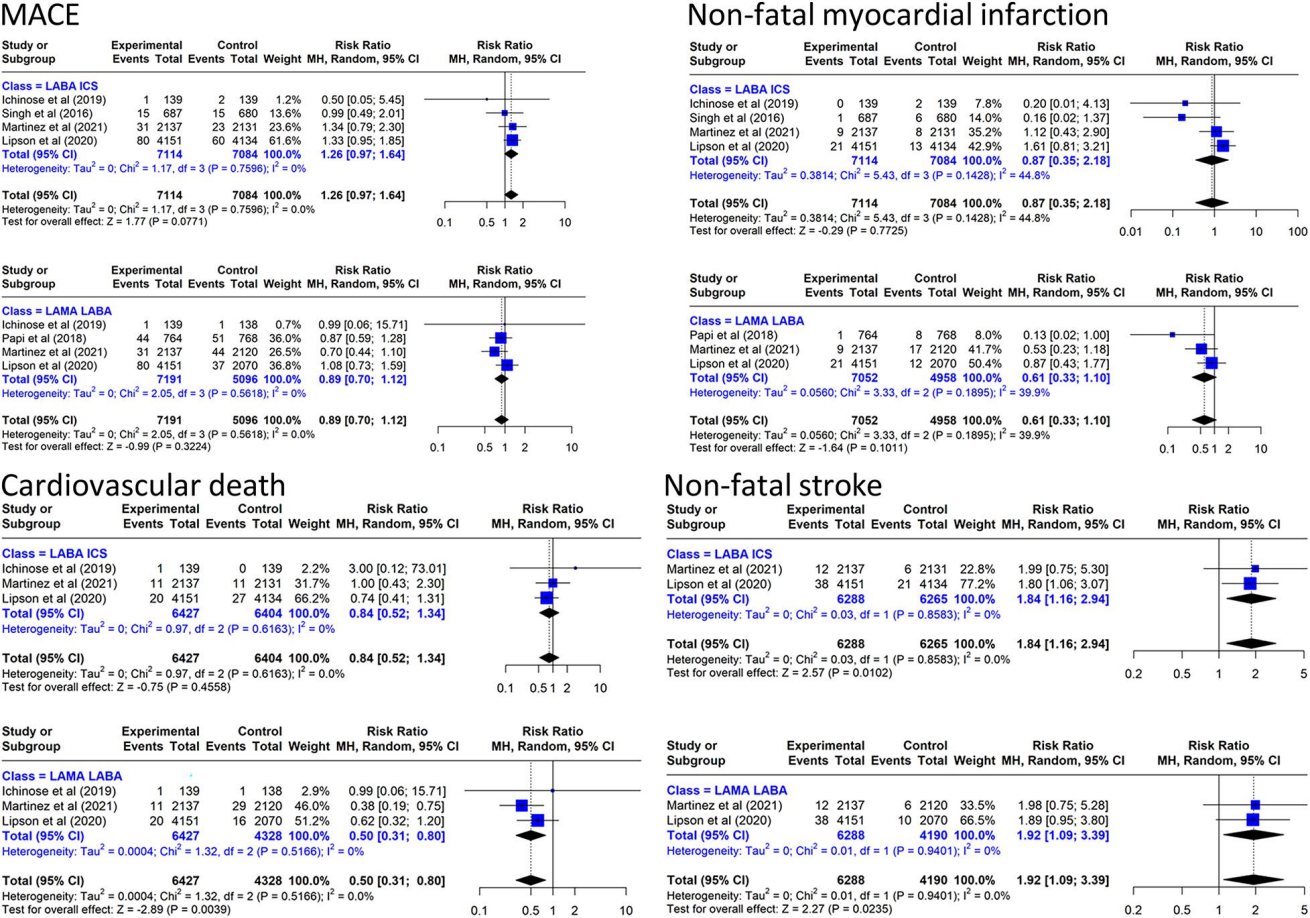
Effect on MACE and components of triple inhaled therapy compared with dual inhaled therapy subtypes. MACE, non-fatal myocardial infarction, cardiovascular death, non-fatal stroke.

The incidence of MACE in the TT group was 21.7/1,000 person/year compared with 14.1/1,000 person/year in the LABA/ICS group and 26.1/1,000 person/year in the LAMA/LABA group. The results of the meta-analysis are presented in Figure 3. Triple inhaled therapy reduced MACE by a non-significant 11% vs. LAMA/LABA (RR: 0.89, 95% CI: 0.70–1.12, four RCTs, *I*^2^ = 0%) and increased it by a non-significant 26% vs. LABA/ICS (RR: 1.26, 95% CI: 0.97–1.64, four RCTs, *I*^2^ = 0%).

In the analysis of MACE components, TT reduced cardiovascular mortality by 50% vs. LAMA/LABA (RR: 0.50, 95% CI: 0.31–0.80, three RCTs, *I*^2^ = 0%) and by a non-significant 16% vs. LABA/ICS (Figure 3). The incidence of cardiovascular deaths in the TT group was 5.93/1,000 person/year compared with 6.64/1,000 person/year in the LABA/ICS group and 11.63/1,000 person/year in the LAMA/LABA group. In the NMA combining direct evidence (three studies), the *P*-score showed that TT ranked first (81%), LABA/ICS ranked second (58%), and LAMA/LABA ranked last (11%) (Supplementary Appendix S6). We performed the same meta-analysis for the off-treatment data without finding clinically relevant differences (Supplementary Appendix S7).

With regard to the other two components of the MACE, TT reduced non-fatal myocardial infarction by 39% (RR: 0.61, 95% CI: 0.33–1.10, four RCTs, *I*^2^ = 39.9%) compared with LAMA/LABA. However, TT increased the risk of non-fatal stroke by 92% (RR: 1.92, 95% CI: 1.09–3.39, two RCTs, *I*^2^ = 0%) compared with LAMA/LABA and by 84% (RR: 1.84, 95% CI: 1.16–2.94, two RCTs, *I*^2^ = 0%) compared with LABA/ICS.

#### Other cardiovascular adverse events

**Figure 4:**
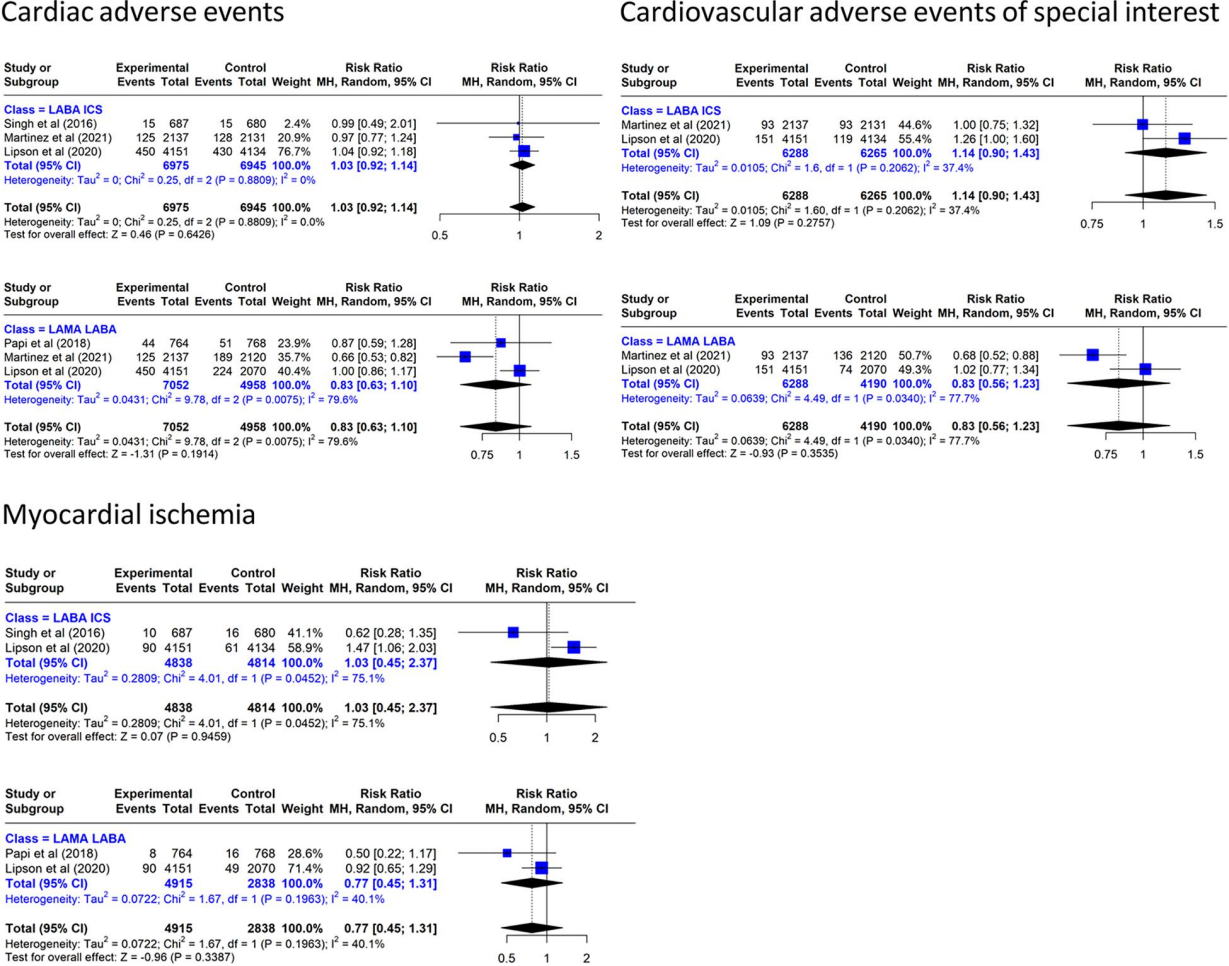
Effect on cardiovascular events of triple inhaled therapy compared with dual inhaled therapy subtypes. Cardiac adverse events, cardiovascular adverse events of special interest, myocardial ischemia.

Triple inhaled therapy reduced CAE by 17% vs. LAMA/LABA (RR: 0.83, 95% CI: 0.63–1.10, three RCTs, *I*^2^ = 79.6%) and increased it by 3% vs. LABA/ICS (RR: 1.03, 95% CI: 0.92–1.14, three RCTs, *I*^2^ = 0%) (Figure 4). With regard to CVAESI, the meta-analysis of two studies (7, 8) showed that TT reduced it by 17% vs. LAMA/LABA (RR: 0.83, 95% CI: 0.56–1.23, two RCTs, *I*^2^ = 77.%) and increased it by 14% vs. LABA/ICS (RR: 1.14, 95% CI: 0.90–1.43, two RCTs, *I*^2^ = 0%) (Figure 4).

In the analysis of myocardial ischemia, TT reduced the incidence by 23% vs. LAMA/LABA (RR: 0.77, 95% CI: 0.45–1.31, two RCTs, *I*^2^ = 40.1%) and increased it by 3% vs. LABA/ICS (RR: 1.03, 95% CI: 0.45–2.37, two RCTs, *I*^2^ = 75.1%).

#### Heart failure

**Figure 5:**
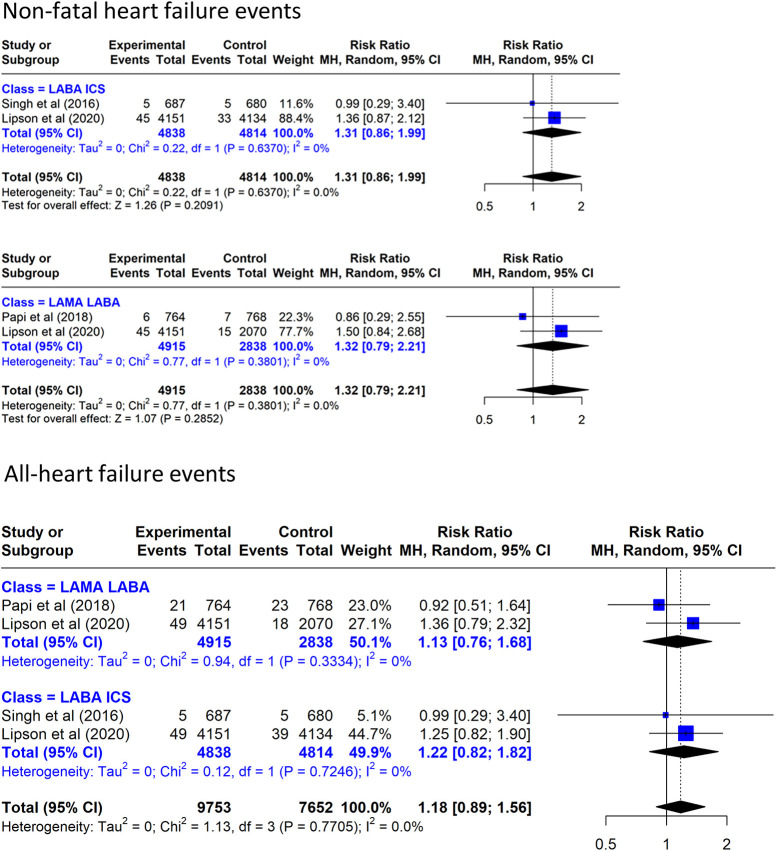
Effect on heart failure events of triple inhaled therapy compared with dual inhaled therapy subtypes. Non-fatal heart failure events, all heart failure events.

TT increased non-fatal HF by a non-significant 32% vs. LAMA/LABA and increased by a non-significant 31% vs. LABA/ICS (Figure 5). When all cases of HF are considered, TT increased all HF by a non-significant 13% vs. LAMA/LABA and increased it by a non-significant 22% vs. LABA/ICS.

#### Sensitivity analysis

The hierarchical ranking for cardiovascular mortality based on *P*-score was not consistent with SUCRA, as LABA/ICS ranked first (86%), triple therapy ranked second (63%), and LAMA/LABA ranked last (10%) (Supplementary Appendix S8). The standard meta-analytic results were not sensitive to this risk of bias in the included studies (Supplementary Appendix S9).

## Discussion

To date, our meta-analysis is the first to specifically analyze the effect of triple inhaled therapy compared with dual inhaled therapy on cardiovascular events in patients with moderate to very severe COPD. TT reduces non-significantly the incidence of MACE compared with LAMA/LABA, but individual analysis of MACE shows that TT significantly reduces cardiovascular mortality compared with LAMA/LABA, with no significant difference compared with LABA/ICS. Similarly, no difference in the incidence of non-fatal myocardial infarction and an increase in non-fatal stroke were found between TT and either of the two dual therapies. These results are particularly interesting because they suggest new hypotheses about the possible effects and mechanisms of TT on cardiovascular events.

Patients with COPD have a high cardiovascular risk due to multifactorial causes (20). The results of the present meta-analysis show a non-significant benefit on MACE of TT compared with both LAMA/LABA and LABA/ICS but show a 50% reduction in cardiovascular mortality compared with LAMA/LABA. This result is very similar to that obtained by Korai et al. (21) on cardiovascular death (RR: 0.50, 95% CI: 0.30–0.80). Another meta-analysis (22) found that LAMA/LABA compared with TT increased the risk of cardiovascular mortality by 91% (RR: 1.91, 95% CI: 1.23–2.99) and the risk of MACE by a non-significant 19% (RR: 1.19, 95% CI: 0.82–1.71). Recently, another meta-analysis confirms that TT reduces cardiovascular mortality compared with LAMA/LABA by 44% (HR 0.46; 95% CI: 0.29–0.71) (23), a result very similar to that obtained in our study. However, none of the meta-analyses cited above were designed to specifically analyze the effect of TT compared with the two classes of dual therapy on cardiovascular events including HF.

It should be noted that non-fatal myocardial infarction and non-fatal stroke are pathophysiologically related to atherosclerotic disease (24, 25). Atherosclerosis is a first-order cardiovascular risk factor, which, however, has a slow and progressive evolution related to the development of atherosclerotic plaque (26). Evidence shows that protection of atherosclerotic plaque is a slow process requiring long-term treatment, so that benefits do not begin to develop before 12–15 months (27, 28). In the present meta-analysis, the included clinical trials had a follow-up period of 52 weeks, which may partly explain the non-significant beneficial effect on non-fatal myocardial infarction. It is also important to consider that inflammation is one of the mechanisms of atherosclerotic plaque development (29–31) and that the potential antiatherosclerotic benefit of TT lies mainly in ICS and their effect on low-intensity systemic inflammation of COPD (32–34). The effect of ICS as systemic anti-inflammatory agents for only 52 weeks could be an additional reason to explain the partial effect of TT on non-fatal myocardial infarction and consequently on MACE. This hypothesis may be compatible with the results of the TORCH study (35), where a 3-year follow-up with the combination of salmeterol with fluticasone showed a lower probability of ischemic cardiovascular events compared with salmeterol alone (11.3% vs. 14.6%, respectively).

The exploration of other cardiovascular variables related to coronary ischemia in this meta-analysis contributes to supporting the possible benefit of TT on coronary atherosclerotic disease. Although they do not reach statistical significance, TT shows a favorable trend in all cases of CAE, myocardial ischemia, and CVAESI compared with LAMA/LABA, with a neutral trend compared with LABA/ICS. These results could support the possible benefit of ICS on coronary atherosclerosis, although the high heterogeneity between comparative studies must be taken into consideration.

The results of the present meta-analysis show that TT increases the incidence of non-fatal stroke compared with both LAMA/LABA and LABA/ICS. These results are similar to those reported in other meta-analyses (22) and are difficult to explain, as it is also an atherosclerotic complication (24, 36). In some of the studies, the classification of stroke is not clearly defined (8, 11), and in some cases, it includes hemorrhagic stroke or thromboembolic stroke (10), which could be confounding factors. Moreover, COPD is associated with a higher risk of major bleeding and a higher risk of atrial fibrillation (AF) (37). The low incidence of events and the low level of evidence according to the GRADE methodology are other factors that call into question this effect. Consequently, the certainty of evidence for non-fatal stroke is low, and the finding should be interpreted cautiously. This outcome should be clarified with specifically designed studies.

The results of the present meta-analysis, where TT compared with LAMA/LABA reduces cardiovascular mortality without reducing myocardial infarction or stroke, suggest the exploration of other beneficial mechanisms. Cardiovascular mortality is mainly related to ischemic heart disease, stroke, HF, and arrhythmias (38, 39). The results of our meta-analysis did not show significant differences between TT and any of the DT subtypes in the incidence of all HF or non-fatal HF. It is therefore possible to suggest that serious arrhythmias could have an impact on the reduction in cardiovascular mortality by TT compared with LAMA/LABA. AF is common in patients with COPD (40), and there is sufficient evidence on the deleterious effect of COPD on AF and cardiovascular mortality (41). It is known that the pulmonary compliance inherent to COPD affects cardiac function and predisposes to arrhythmias (3, 42, 43). It might therefore be thought that the simultaneous use of LAMA, LABA, and ICS could have a more effective effect in reducing pulmonary compliance (44, 45) and secondarily reduce the risk of arrhythmias and therefore cardiovascular mortality. In addition, TT compared with DT reduces severe exacerbations that predispose to severe arrhythmias (46). In the present meta-analysis, we were unable to meta-analyze the incidence of arrhythmias due to the lack of data. Therefore, specific studies are necessary to clarify the relationship between TT and arrhythmias and the potential benefit of TT compared with DT on malignant arrhythmias.

Although it was not the main objective of the present meta-analysis, the LABA/ICS combination shows a non-significantly different effect compared with TT on the main cardiovascular variables analyzed. For this reason, we compared LABA/ICS vs. LAMA/LABA observing a significant reduction in the incidence of non-fatal myocardial infarction and CVAESI and a clearly favorable trend in the incidence of MACE and cardiovascular mortality. Similar results are observed in other meta-analyses, where LABA/ICS reduces the incidence of MACE by 42% and that of myocardial infarction by 77% compared with LAMA/LABA (22). Since a meta-analysis of 219 studies involving 228,710 patients found that ICS did not increase the incidence of fatal pneumonia (47), we consider it appropriate to consider the LABA/ICS combination as an option to reduce all-cause mortality and cardiovascular events. The subsequent association of LAMA could improve lung function parameters and reduce exacerbations without increasing cardiovascular death (48, 49).

## Conclusion

Triple inhaled therapy compared with dual inhaled therapy with LAMA/LABA shows a non-significant favorable trend in reducing MACE, and a significant reduction in cardiovascular mortality in patients with moderate to very severe COPD and previous exacerbations. The results also show a possible increase in the incidence of non-fatal stroke, although with a low level of evidence. Future specifically designed studies with long-term follow-up should confirm these results as well as the potential effect of triple inhaled therapy on fatal arrhythmias and sudden death as factors involved in cardiovascular mortality.

### Strengths and weaknesses

The main strength of the meta-analysis is that it evaluated the effect of TT compared with the two subtypes of DT in the same populations, thus avoiding selection bias in the patients included in the different RCTs. To analyze MACE and cardiovascular mortality, unlike other meta-analyses, we preferred to use on-treatment outcomes rather than off-treatment outcomes to analyze the efficacy of TT. Nevertheless, we also analyzed off-treatment data without finding relevant differences. We only included studies with a 12-month follow-up to ensure a clinically relevant incidence of cardiovascular events. The results show low heterogeneity among the included studies, and most of the results correspond to classical meta-analytic statistical techniques.

In terms of weaknesses, the study has some limitations. First, the definition of variables is not uniform between different studies. To minimize this bias, unlike other meta-analyses, we used the data from the restricted definition of MACE from the IMPACT study to obtain more homogeneous and reproducible results. The absence of objective protocolized data for event adjudication should be taken into consideration when interpreting the results. The statistical results should be interpreted in the context of a low incidence of cardiovascular events with some cases that could not be adjudicated. Another aspect to consider is that the number of studies included is small, and two of them account for 80% of the sample, although the low heterogeneity lends consistency to the results. Furthermore, it is not possible from our analysis to predict which patients might have a greater benefit from TT in the population studied. Finally, the results should be interpreted for the moderate and very severe COPD population with previous exacerbations.

### Implications

Although TT compared with DT showed only a non-significant favorable trend in reducing MACE, it reduced cardiovascular mortality by 50% compared with LAMA/LABA and should be considered as a primary treatment option when the goal is to reduce cardiovascular death in COPD patients. Furthermore, the LABA/ICS combination has cardiovascular benefits compared with LAMA/LABA and could therefore be considered a reasonable alternative in patients with COPD and high cardiopulmonary risk. The present meta-analysis should be updated when the ongoing trial (12) is completed, and there is a need to perform a NMA.

## Data Availability

The datasets presented in this study can be found in online repositories. The names of the repository/repositories and accession number(s) can be found in the article/Supplementary Material.
