# Supplementary material for: Effect of triple inhaled therapy on MACE and cardiovascular events in COPD: a systematic review and meta-analysis
Source: Front Cardiovasc Med. 2025 Dec 4;12:1680080. doi: 10.3389/fcvm.2025.1680080 (PMC12711773; doi:10.3389/fcvm.2025.1680080)

Supplement

1.Appendix S1. Final Search strings and terms selected for the different databases and the citations retrieved.

2.Appendix S2. Descriptive table of study selection for the systematic review of inhaled therapy in Chronic Obstructive Pulmonary Disease

3.Appendix 3. Selection and definition of outcomes

4.Appendix 4. Meta-analysis of the comparison of LABA/ICS with LAMA/LABA for MACE and its components

5.Appendix S5. Assessment of risk of bias (ROB2) by domain in the studies included in the meta-analysis

6.Appendix S6. Network meta-analysis for cardiovascular mortality

7.Appendix S7. Meta-analysis for MACE and its components using off-treatment data

8.Appendix S8. Sensitivity analysis

9.Appendix S9. Funnel plots to detect potential reporting biases on cardiovascular mortality

Appendix S1. Final Search strings and terms selected for the different databases and the citations retrieved.

PUBMED: Citations: 299

(((Chronic obstructive pulmonary disease[Title/Abstract]) OR (copd[Title/Abstract])) AND (mortality[Title/Abstract]) AND ((inhalated therapy[Title/Abstract]) OR (inhaled therapy[Title/Abstract]) OR (dual[Title/Abstract]) OR (triple[Title/Abstract]))) AND (trial)

SCOPUS: Citations: 208

TITLE-ABS-KEY ((chronic AND obstructive AND pulmonary AND disease OR copd AND mortality AND inhaled AND therapy OR inhaled AND therapy OR dual AND therapy OR triple AND therapy AND clinical AND randomised AND trials))

EMBASE: Citations: 272

(('chronic obstructive lung disease'/exp OR 'chronic obstructive lung disease' OR chronic) AND obstructive AND pulmonary AND ('disease'/exp OR disease) AND ('mortality'/exp OR mortality) AND inhaled AND ('therapy'/exp OR therapy) OR inhalated) AND ('therapy'/exp OR therapy) AND ('clinical'/exp OR clinical) AND trials

Appendix S2. Descriptive table of study selection for the systematic review of inhaled therapy in Chronic Obstructive Pulmonary Disease

		n	Exclusion	Number of studies excluded for reasons		Remaining
Identification	PubMed<br>Scopus<br>Embase<br>Expert provided	299<br>208<br>272<br>2				781
Screening	Duplicates		414	Duplication		367
	Tittle/abstract assessed for elegibility	367	350	Reviews and meta-analyses<br>No randomized clinical trials<br>No intervention<br>Post hoc analysis<br>RCT no included TT vs DT comparations<br>Others	47<br>148<br>79<br>11<br>28<br>37	17
	Full-text citations assessed for elegibility	17	12	Sub-analysis<br>No cardiovascular/all mortality outcomes<br>Treatment duration < 12 months<br>Missing data	3<br>6<br>2<br>1	5
Included	Included	5	Triple inhaled therapy vs LAMA/LABA 4<br>Triple inhaled therapy vs LABA/ICS 4			

TT: triple inhaled therapy. DT: Dual inhaled therapy. RCT: Randomized clinical trial. LAMA: long-acting muscarinic antagonist. LABA: long-acting β2-agonist ICS: inhaled glucocorticoids

Appendix 3. Selection and definition of outcomes

In all studies, strict and objective criteria for the diagnosis of the different outcomes (MeDRa) were not followed. In none of the studies were objective diagnostic tests required. Adjudication committees for cardiovascular events existed in 80% of the studies.

Whenever available, on-treatment data were used for the main analysis. An additional exploratory meta-analysis was performed with the off-treatment data from ETHOS and IMPACT for MACE and its components

MACE: Major Adverse Cardiovascular Events including cardiovascular death (CVD), non-fatal myocardial infarction and non-fatal stroke. For the ETHOS study the original database was used and for the IMPACT study the original data from the strict (non-expanded) MACE were used. The KRONOS study did not clearly define MACE. The TRILOGY study included myocardial infarction, stroke, arrhythmias and heart failure. The TRIBUTE study considered MACE as cardiac adverse events.

Cardiovascular death (CVD)/Cardiovascular mortality: TRILOGY and TRIBUTE data were not available. On treatment data were used in the main analysis. Data used from ETHOS and IMPACT correspond to the databases reviewed.

Non-fatal myovcardial infarction: In all five studies were recorded individually with the strict definition.

Non-fatal stroke: They were recorded with the literally definition in ETHOS, IMPACT and KRONOS. However, IMPACT included both thromboembolic and haemorrhagic stroke episodes within the definition and the other two studies did not clearly define them.TRILOGY and TRIBUTE have no data available.

Myocardial ischaemia: TRIBUTE and TRILOGY defined it literally. For IMPACT, the sum of non-fatal myocardial infarction and non-acute ischaemia is used. ETHOS and KRONOS had no data available.

Cardiovascular adverse events of special interest (CVAESI): ETHOS data correspond to the revised database. IMPACT data used correspond to serious CVAESI.

Cardiac adverse events (CAE): Defined as such in TRILOGY, TRIBUTE, ETHOS and IMPACT. KRONOS has no data available.

Appendix S4. Meta-analysis of the comparison of LABA/ICS with LAMA/LABA for MACE and its components

LABA/ICS as experimental outcome and LAMA/LABA as control

Mayor cardiovascular events (MACE)

Study	Experimental Events	Experimental Total	Control Events	Control Total	Weight	Risk Ratio MH, Random, 95% CI
Ichinose et al (2019)	2	139	1	138	2.9%	1.99 [0.18; 21.65]
Martinez et al (2021)	23	2131	44	2120	42.8%	0.52 [0.32; 0.86]
Lipson et al (2020)	60	4134	37	2070	54.3%	0.81 [0.54; 1.22]
**Total (95% CI)**		**6404**		**4328**	**100.0%**	**0.69 [0.45; 1.04]**

Heterogeneity: $Tau^2 = 0.0397$; $Chi^2 = 2.59$, df = 2 (P = 0.2734); $I^2 = 22.9\%$
Test for overall effect: Z = -1.76 (P = 0.0786)

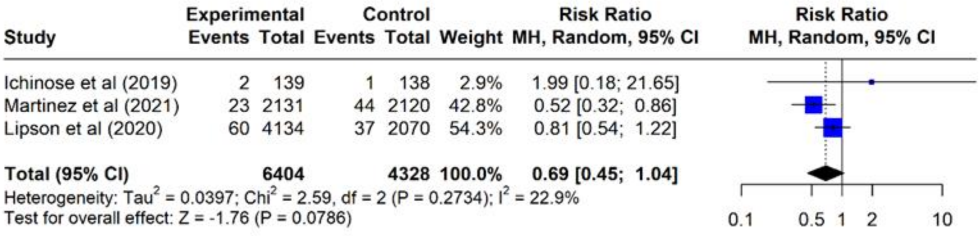

Cardiovascular death

Study	Experimental Events	Experimental Total	Control Events	Control Total	Weight	Risk Ratio MH, Random, 95% CI
Ichinose et al (2019)	2	139	1	138	1.0%	1.99 [0.18; 21.65]
Martinez et al (2021)	46	2131	64	2120	38.9%	0.72 [0.49; 1.04]
Lipson et al (2020)	109	4134	66	2070	60.1%	0.83 [0.61; 1.12]
**Total (95% CI)**		**6404**		**4328**	**100.0%**	**0.79 [0.62; 1.00]**

Heterogeneity: $Tau^2 = 0$; $Chi^2 = 0.93$, df = 2 (P = 0.6273); $I^2 = 0.0\%$
Test for overall effect: Z = -2.00 (P = 0.0454)

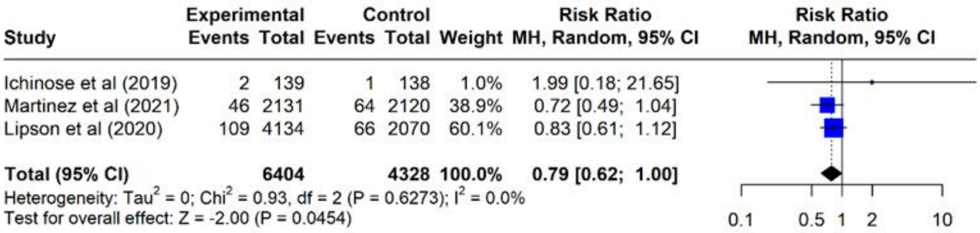

Non-fatal myocardial infarction

Study	Experimental Events	Experimental Total	Control Events	Control Total	Weight	Risk Ratio MH, Random, 95% CI
Martinez et al (2021)	8	2131	17	2120	46.6%	0.47 [0.20; 1.08]
Lipson et al (2020)	13	4134	12	2070	53.4%	0.54 [0.25; 1.19]
**Total (95% CI)**		**6265**		**4190**	**100.0%**	**0.51 [0.29; 0.90]**

Heterogeneity: $Tau^2 = 0$; $Chi^2 = 0.06$, df = 1 (P = 0.8013); $I^2 = 0.0\%$
Test for overall effect: Z = -2.33 (P = 0.0198)

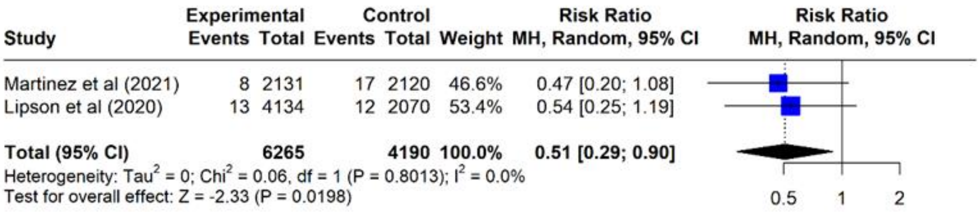

Non-fatal stroke

Study	Experimental Events	Experimental Total	Control Events	Control Total	Weight	Risk Ratio MH, Random, 95% CI
Martinez et al (2021)	6	2131	6	2120	30.6%	0.99 [0.32; 3.08]
Lipson et al (2020)	21	4134	10	2070	69.4%	1.05 [0.50; 2.23]
**Total (95% CI)**		**6265**		**4190**	**100.0%**	**1.03 [0.55; 1.93]**

Heterogeneity: $Tau^2 = 0$; $Chi^2 = 0.01$, df = 1 (P = 0.9362); $I^2 = 0.0\%$
Test for overall effect: Z = 0.10 (P = 0.9170)

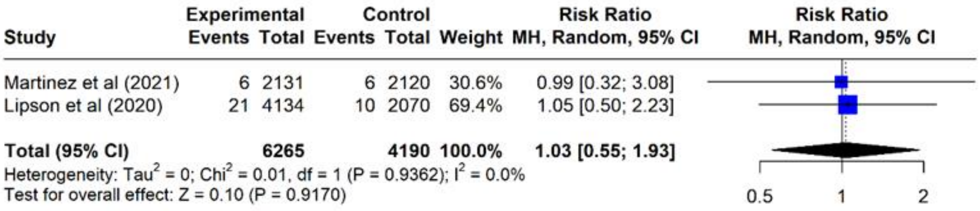

Appendix S5. Assessment of risk of bias (ROB2) by domain in the studies included in the meta-analysis

					BIAS ARISING FROM THE RANDOMIZATION PROCESS (R)	BIAS DUE TO DEVIATIONS FROM INTENDED INTERVENTIONS (D)	BIAS DUE TO MISSING OUTCOME DATA (MI)	BIAS IN MEASUREME[illegible] OUTCOME (M[illegible]
Study name	Authors	First Author	Year	Journal	Risk of bias judgement	Risk of bias judgement	Risk of bias judgement	Risk of bias judg[illegible]
KRONOS (extension)	Ichinose M, Fukushima Y, Inoue Y, Hataji O, Ferguson GT, Rabe KF, Hayashi N, Okada H, Takikawa M, Bourne E, Ballal S, DeAngelis K, Aurivillius M, Reisner C, Dorinsky P.	Ichinose M	2019	Int J Chron Obstruct Pulmon Dis. 2019 Dec 23;14:2993-3002. doi: 10.2147/COPD.S220861.	low	low	low	some concern[illegible]
TRIBUTE	Papi A, Vestbo J, Fabbri L, Corradi M, Prunier H, Cohuet G, Guasconi A, Montagna I, Vezzoli S, Petruzzelli S, Scuri M, Roche N, Singh D	Papi A	2018	Lancet. 2018 Mar 17;391(10125):1076-1084. doi: 10.1016/S0140-6736(18)30206-X. Epub 2018 Feb 9.	low	low	high	low
TRILOGY	Singh D, Papi A, Corradi M, Pavlišová I, Montagna I, Francisco C, Cohuet G, Vezzoli S, Scuri M, Vestbo J.	Singh D	2016	Lancet. 2016 Sep 3;388(10048):963-73. doi: 10.1016/S0140-6736(16)31354-X. Epub 2016 Sep 1	low	low	high	low
ETHOS	Martinez FJ, Rabe KF, Ferguson GT, Wedzicha JA, Singh D, Wang C, Rossman K, St Rose E, Trivedi R, Ballal S, Darken P, Aurivillius M, Reisner C, Dorinsky P	Martinez FJ	2021	Am J Respir Crit Care Med. 2021 Mar 1;203(5):553-564. doi: 10.1164/rccm.202006-2618OC.	low	low	low	low
IMPACT	Lipson DA, Crim C, Criner GJ, Day NC, Dransfield MT, Halpin DMG, Han MK, Jones CE, Kilbride S, Lange P, Lomas DA, Lettis S, Manchester P, Martin N, Midwinter D, Morris A, Pascoe SJ, Singh D, Wise RA, Martinez FJ	Lipson DA	2020	Am J Respir Crit Care Med. 2020 Jun 15;201(12):1508-1516. doi: 10.1164/rccm.201911-2207OC.	low	low	low	low

Support for judgement : RoB 2: A revised Cochrane risk-of-bias tool for randomized trials. https://methods.cochrane.org/bias/resources/rob-2-revised-cochrane-risk-bias[illegible]

Appendix 6. Network meta-analysis for cardiovascular mortality and all-cause mortality (results display)

Frequentist Analysis

Number of studies: k = 3

Number of pairwise comparisons: m = 7

Number of observations: o = 14908

Number of treatments: n = 3

Number of designs: d = 2

Common effects model (Mantel-Haenszel method)

Treatment estimate (sm = 'OR'):

	LABA_ICS	LAMA_LABA	LAMA_LABA_ICS
LABA_ICS	…..	0.7476	1.1171
LAMA_LABA	1.3376	….	1.4943
LAMA_LABA_ICS	0.8952	0.6692	….

Lower 95%-confidence limit:

	LABA_ICS	LAMA_LABA	LAMA_LABA_ICS
LABA_ICS	….	0.4338	0.7187
LAMA_LABA	0.7762	….	0.8583
LAMA_LABA_ICS	0.5759	0.3844	….

Upper 95%-confidence limit:

	LABA_ICS	LAMA_LABA	LAMA_LABA_ICS
LABA_ICS	….	1.2884	1.7364
LAMA_LABA	2.3052	….	2.6015
LAMA_LABA_ICS	1.3914	1.1651	….

Test of inconsistency (between designs):

Q d.f. p-value

0.09 1 0.7665

League table (common effects model):

LABA_ICS	0.75 (0.43; 1.32)	1.12 (0.72; 1.74)	0.75 (0.43; 1.29)
LAMA_LABA	1.52 (0.86; 2.70)	1.12 (0.72; 1.74)	1.49 (0.86; 2.60)
LAMA_LABA_ICS			

P-score

LAMA_LABA_ICS	0.8054
LABA_ICS	0.5819
LAMA_LABA	0.1126

Bayesian Analysis

Gelman Statistic 1.000658

Rank probability (SUCRA):

LABA_ICS	0.8645750
LAMA_LABA_ICS	0.6251125
LAMA_LABA	0.0103125

Appendix S7. Meta-analysis for MACE and its components using off-treatment data

MACE

Study or Subgroup	Experimental Events	Experimental Total	Control Events	Control Total	Weight	Risk Ratio MH, Random, 95% CI
**Class = LAMA LABA**						
Ichinose et al (2019)	1	116	1	111	4.2%	0.96 [0.06; 15.11]
Papi et al (2018)	5	764	19	768	20.2%	0.26 [0.10; 0.70]
Martinez et al (2021)	31	2137	44	2120	35.3%	0.70 [0.44; 1.10]
Lipson et al (2020)	133	4151	66	2070	40.3%	1.00 [0.75; 1.34]
**Total (95% CI)**		**7168**		**5069**	**100.0%**	**0.67 [0.37; 1.22]**
Heterogeneity: $Tau^2 = 0.2074$; $Chi^2 = 7.44$, df = 3 (P = 0.0592); $I^2 = 59.7\%$						
**Total (95% CI)**		**7168**		**5069**	**100.0%**	**0.67 [0.37; 1.22]**
Heterogeneity: $Tau^2 = 0.2074$; $Chi^2 = 7.44$, df = 3 (P = 0.0592); $I^2 = 59.7\%$						

Risk Ratio MH, Random, 95% CI

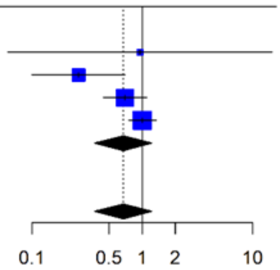

Study or Subgroup	Experimental Events	Experimental Total	Control Events	Control Total	Weight	Risk Ratio MH, Random, 95% CI
**Class = LABA ICS**						
Ichinose et al (2019)	1	116	0	58	0.5%	1.51 [0.06; 36.41]
Singh et al (2016)	15	687	15	680	9.6%	0.99 [0.49; 2.01]
Martinez et al (2021)	31	2137	23	2131	16.7%	1.34 [0.79; 2.30]
Lipson et al (2020)	133	4151	100	4134	73.3%	1.32 [1.03; 1.71]
**Total (95% CI)**		**7091**		**7003**	**100.0%**	**1.29 [1.04; 1.61]**
Heterogeneity: $Tau^2 = 0$; $Chi^2 = 0.61$, df = 3 (P = 0.8940); $I^2 = 0\%$						
**Total (95% CI)**		**7091**		**7003**	**100.0%**	**1.29 [1.04; 1.61]**
Heterogeneity: $Tau^2 = 0$; $Chi^2 = 0.61$, df = 3 (P = 0.8940); $I^2 = 0.0\%$						

Risk Ratio MH, Random, 95% CI

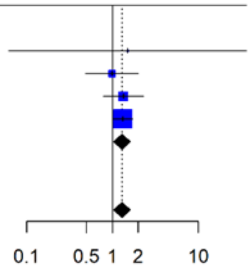

CARDIOVASCULAR MORTALITY (CVD)

Study or Subgroup	Experimental Events	Experimental Total	Control Events	Control Total	Weight	Risk Ratio MH, Random, 95% CI
**Class = LAMA LABA**						
Ichinose et al (2019)	1	116	1	111	3.2%	0.96 [0.06; 15.11]
Martinez et al (2021)	11	2137	29	2120	41.8%	0.38 [0.19; 0.75]
Lipson et al (2020)	26	4151	20	2070	55.0%	0.65 [0.36; 1.16]
**Total (95% CI)**		**6404**		**4301**	**100.0%**	**0.52 [0.32; 0.86]**
Heterogeneity: $Tau^2 = 0.0282$; $Chi^2 = 1.58$, df = 2 (P = 0.4539); $I^2 = 0\%$						
**Total (95% CI)**		**6404**		**4301**	**100.0%**	**0.52 [0.32; 0.86]**
Heterogeneity: $Tau^2 = 0.0282$; $Chi^2 = 1.58$, df = 2 (P = 0.4539); $I^2 = 0.0\%$						

Risk Ratio MH, Random, 95% CI

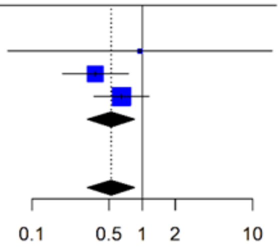

Study or Subgroup	Experimental Events	Experimental Total	Control Events	Control Total	Weight	Risk Ratio MH, Random, 95% CI
**Class = LABA ICS**						
Ichinose et al (2019)	1	116	0	58	1.9%	1.51 [0.06; 36.41]
Martinez et al (2021)	11	2137	11	2131	27.4%	1.00 [0.43; 2.30]
Lipson et al (2020)	26	4151	31	4134	70.7%	0.84 [0.50; 1.40]
**Total (95% CI)**		**6404**		**6323**	**100.0%**	**0.89 [0.57; 1.37]**
Heterogeneity: $Tau^2 = 0$; $Chi^2 = 0.23$, df = 2 (P = 0.8898); $I^2 = 0\%$						
**Total (95% CI)**		**6404**		**6323**	**100.0%**	**0.89 [0.57; 1.37]**
Heterogeneity: $Tau^2 = 0$; $Chi^2 = 0.23$, df = 2 (P = 0.8898); $I^2 = 0.0\%$						

Risk Ratio MH, Random, 95% CI

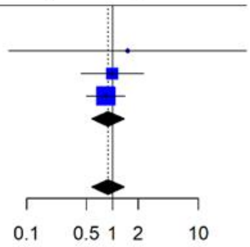

Appendix S8. Sensitivity analysis

Meta-analysis of cardiovascular mortality. No difference from the full analysis shown in the original article.

Cardiovascular mortality

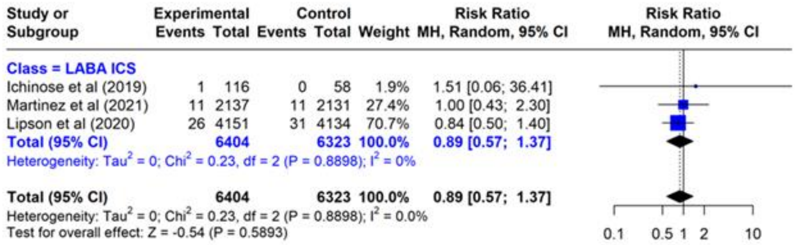

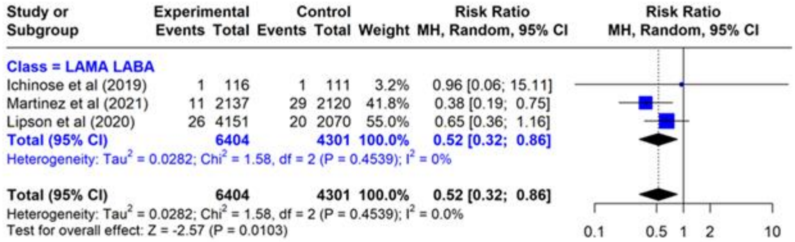

Appendix S9. Funnel plots to detect potential reporting biases on cardiovascular mortality and all-cause mortality

Cardiovascular mortality

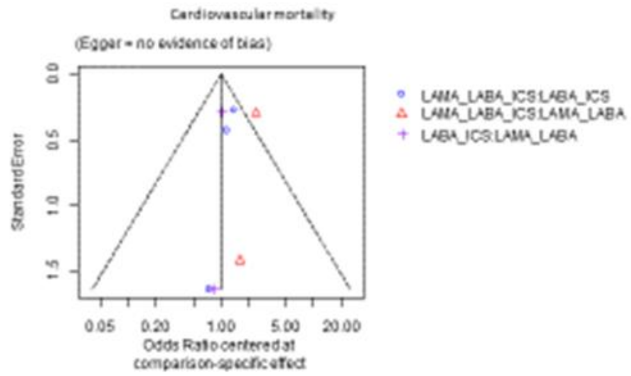

Supplement: Supplementary file 1 [file Datasheet1.pdf]
